# A Human Dectin-2 Deficiency Associated With Invasive Aspergillosis

**DOI:** 10.1093/infdis/jiab145

**Published:** 2021-03-18

**Authors:** James S Griffiths, P Lewis White, Magdalena A Czubala, Elena Simonazzi, Mariolina Bruno, Aiysha Thompson, Pierre J Rizkallah, Mark Gurney, Diogo M da Fonseca, Julian R Naglik, Wendy Ingram, Keith Wilson, Frank L van de Veerdonk, Rosemary Barnes, Philip R Taylor, Selinda J Orr

**Affiliations:** 1 Division of Infection and Immunity and Systems Immunity Research Institute, Cardiff University School of Medicine, Cardiff, United Kingdom; 2 Centre for Host-Microbiome Interactions, Faculty of Dentistry, Oral and Craniofacial Sciences, King’s College London, London, United Kingdom; 3 Public Health Wales Microbiology Cardiff, University Hospital of Wales, Cardiff, United Kingdom; 4 United Kingdom Dementia Research Institute at Cardiff, Cardiff, United Kingdom; 5 Department of Internal Medicine, Radboud University Medical Center, Nijmegen, The Netherlands; 6 University Hospital of Wales, Cardiff, United Kingdom; 7 Wellcome-Wolfson Institute for Experimental Medicine, School of Medicine, Dentistry and Biomedical Science, Queen’s University Belfast, Belfast, United Kingdom

**Keywords:** Dectin-2, CLR, *Aspergillus*, *Candida*, fungal immunology, host–pathogen interactions, innate immunity, inflammation

## Abstract

Immunocompromised patients are highly susceptible to invasive aspergillosis. Herein, we identified a homozygous deletion mutation (507 del C) resulting in a frameshift (N170I) and early stop codon in the fungal binding Dectin-2 receptor, in an immunocompromised patient. The mutated form of Dectin-2 was weakly expressed, did not form clusters at/near the cell surface and was functionally defective. Peripheral blood mononuclear cells from this patient were unable to mount a cytokine (tumor necrosis factor, interleukin 6) response to *Aspergillus fumigatus*, and this first identified Dectin-2–deficient patient died of complications of invasive aspergillosis.

## Teaser

We identified a Dectin-2 N170I mutation in an immunocompromised patient who died of complications of invasive aspergillosis. This mutation results in an early stop codon and poor receptor expression and renders Dectin-2 functionally defective.

Invasive fungal infections including invasive aspergillosis (IA) represent a severe disease burden in immunocompromised patients such as acute myeloid leukemia patients and allogeneic hematopoietic stem cell transplant (HSCT) recipients. Absence of a robust antifungal immune response permits fungal colonization, invasive growth, and disease. IA mortality is unacceptably high (30%–80%) in allogeneic HSCT recipients [1, 2]. Therefore, patients are empirically treated with antifungal therapies.

The C-type lectin-like receptors (CLRs) Dectin-1 and Dectin-2 drive antifungal immune responses against *Candida* and *Aspergillus* [3–7]. A Dectin-1 single-nucleotide polymorphism (SNP) (Y238X) has been associated with mucocutaneous candidiasis [8] and increased susceptibility to IA [9]. SNPs in Dectin-2 have been associated with IA [10]; however, their functional consequences are unknown. Herein, we characterized a novel Dectin-2 exonic deletion mutation identified in an immunocompromised patient who died of complications of IA.

## MATERIALS AND METHODS

### Ethics Statement

The study was approved by the National Institute for Social Care and Health Research Ethics Committee (reference number 14/WA/1119). Written informed consent was obtained from patients in the study. Animal work was performed according to institutional and United Kingdom (UK) Home Office guidelines. This study was performed in accordance with the Project License. Procedures were approved by the Cardiff University Animal Welfare and Ethical Review Body and UK Home Office. The animal care and use protocol adhered to the Animals (Scientific Procedures) Act 1986.

### Genetic Analysis

RNA was extracted from patient blood samples using PAXgene Blood RNA kit (Qiagen), and complementary DNA (cDNA) was generated using a Reverse Transcription Kit (Thermo Fisher Scientific). Dectin-2 DNA was amplified and sequenced from patient cDNA by polymerase chain reaction (PCR) using primers (Supplementary Table 1).

### Structural Analysis

The structure of wild-type (WT) Dectin-2, Protein Data Bank accession code 5VYB, was used as the starting model. The Coot program was used to implement mutations and model readjustment. REFMAC5 (CCP4) was used to regularize model geometry.

### Cloning and Transfection

WT and mutant *CLEC6A*/Dectin-2-pFB-NEO (Stratagene) and pHR’SIN-cPPT-SXW (pSXW) constructs, with an N-terminus FLAG-tagged Dectin-2, were generated using primers (Supplementary Table 1). FLAG-tagged Dectin-2 WT and mutant PCR products were inserted into pFB-NEO using the In-Fusion Cloning Kit (Clontech). Stellar Competent Cells (Clontech) were transformed with *CLEC6A*/Dectin-2-FLAG-Tag constructs and grown in LB broth (Sigma) before plasmid DNA was extracted using the DNA Mini/Midiprep Kit (Thermo Fisher Scientific/Qiagen). FLAG-tagged Dectin-2 WT and mutant PCR products were inserted into SXW lentiviral vector using the In-Fusion Cloning Kit. Top10 competent *Escherichia coli* (NEB) were transformed with *CLEC6A*/Dectin-2-FLAG-Tag constructs and grown in LB broth before plasmid DNA was extracted using the DNA Miniprep Kit. HEK293T cells cultured in Dulbecco’s modified Eagle’s medium (DMEM) (with 10% fetal bovine serum [FBS], 100 U/mL penicillin/streptomycin [Thermo Fisher Scientific]) were co-transfected with 1.5 µg FcγR pMXs-IP, and either pFB-NEO or pFB-NEO containing WT or mutant Dectin-2, using Fugene-6 Transfection Reagent (Promega). Forty-eight hours later, cells were harvested for RNA/protein analysis.

### Lentivirus Infection

HEK293T cells cultured in DMEM medium with 10% FBS and 100 U/mL penicillin/streptomycin were co-transfected with 1.5 µg pR8.91, 1 µg pMD2G, and either pSXW empty vector, Dectin-2 WT pSXW, or Dectin-2 N107I pSXW, using Effectene Transfection Reagent (Qiagen). Forty-eight hours and 72 hours later, supernatant was collected, filtered using 0.45-µm sterile millexGP filter (Millipore Ireland Ltd) and overlaid on 20% sucrose (Sigma) gradient in ultracentrifuge conical tubes (Beckman Coulter). The gradient was centrifuged at 120 000*g* at 4°C for 90 minutes and virus pellet resuspended in AIM V medium (Thermo Fisher Scientific). Bone marrow (BM) cells were isolated from Dectin-1–Dectin-2 (D1D2) double knockout (DKO) mice. BM-derived macrophages (BMDMs) were generated as previously described [7]. For lentiviral infection, BMDMs were harvested and lentivirus was added in the presence of fresh media containing macrophage colony-stimulating factor.

### Determination of Dectin-2 Expression

Forty-eight hours after transfection of HEK293T cells and 72 hours after infection of D1D2 DKO BMDMs, RNA was extracted using TRIZOL (Thermo Fisher Scientific) and purified using the RNeasy Mini Kit (Qiagen). cDNA was synthesized using the TaqMan Reverse Transcription Kit (Invitrogen). *CLEC6A*/Dectin-2 mRNA was quantified by quantitative PCR using ABI Taqman Primer/Probe Sets (Thermo Fisher Scientific) and normalized against *HPRT1*. Dectin-2 protein expression was measured by intracellular flow cytometry staining with anti-FLAG (L5 BioLegend) or by surface flow cytometry staining with anti–Dectin-2 (545943 R&D).

### Cytospin

Seventy-two hours after lentiviral infection, D1D2 DKO BMDMs were collected and centrifuged. The pellet was fixed with BD Cytofix/Cytoperm solution (BD Biosciences), washed with BD Perm-Wash solution (BD Biosciences), and blocked with flow cytometry block solution (Dulbecco’s phosphate-buffered saline [DPBS], 5 mM ethylenediaminetetraacetic acid, 2 mM sodium azide, 0.5% bovine serum albumin, 5% rabbit serum) containing 4 μg/mL 2.4G2. Cells were stained with anti-CD11b (M1/70 BioLegend) and anti-FLAG (L5 BioLegend) and washed with BD Perm-Wash. DAPI (4’,6-diamidino-2-phenylindole) (Thermo Fisher Scientific) was added before cells were washed and resuspended in DPBS. 10^4^ cells per sample were cytospun and mounted with Prolong Gold Antifade (Fisher Scientific). Cells were imaged using a Zeiss Cell Observer Spinning Disk confocal microscope with a 63× objective to obtain Z-stacks of the whole cell thickness. Images were then analyzed using Imaris 9.3.1 to obtain 3D reconstruction of cell structure.

### Fungal Cultures

*Aspergillus fumigatus* 13073 (American Type Culture Collection [ATCC]) was cultured on potato dextrose agar for 7 days at 37°C. Conidia were harvested and passed through a 40-μM filter to remove hyphal fragments. Resting conidia were washed and resuspended in DPBS [7]. *Candida albicans* SC5314 (ATCC) was cultured on YPD agar plates overnight at 30°C, then cultured in YPD broth for 16 hours at 30°C with shaking, washed with DPBS, and resuspended in DPBS [5].

### Generation of Bone-Marrow–Derived Dendritic Cells

Bone marrow–derived dendritic cells (BMDCs) were generated by culturing BM cells for 8–10 days in RPMI 1640 medium containing 10% FBS, 2 mM l-glutamine (Thermo Fisher Scientific), 100 U/mL penicillin/streptomycin, 10 mM HEPES (Life Technologies), 1% nonessential amino acids solution (Life Technologies), 1 mM sodium pyruvate (Thermo Fisher Scientific), 50 μM β-mercaptoethanol (Fisher), and 10 ng/mL granulocyte macrophage colony-stimulating factor (Peprotech).

### Cytokine Assays

Human peripheral blood mononuclear cells (PBMCs) were isolated from patient blood using FicollPLUS (Sigma), washed with PBS^Mg+Ca+^ (Thermo Fisher Scientific), and washed 3 times with RPMI 1640. Cells were resuspended in PBMC media (RPMI 1640 with 10% FBS, 2% human serum [Sigma], and 10 mM l-glutamine, 10 mM sodium pyruvate, and 100 μg/mL gentamicin [all from Thermo Fisher Scientific]). PBMCs were rested for 4 hours before 100 μL of 5 × 10^6^ PBMCs/mL was challenged with 100 μL of 1 μg/mL lipopolysaccharide (LPS) or 100 μL of 5 × 10^6^/mL *A. fumigatus* swollen conidia. Swollen conidia were cultured in RPMI at 37°C for 6 hours. After 24 hours, supernatant was collected and cytokines were measured by enzyme-linked immunosorbent assay (ELISA) (R&D). WT and Dectin-2 knockout (KO) BMDCs were resuspended in RPMI containing 10% FBS and 100 U/mL penicillin/streptomycin. One hundred microliters of 1 × 10^6^ BMDCs/mL was challenged with 100 μL of 1 × 10^6^/mL *A. fumigatus* conidia, 100 μL of 1 × 10^6^/mL *C. albicans*, or 100 μL of 2 ng/mL LPS. Amphotericin B (2.5 µg/mL) was added to *C. albicans* 2 hours after stimulation. After 24 hours, supernatant was collected, and cytokines measured by ELISA (R&D).

### Statistical Methods

Data were analyzed using GraphPad Prism. Data are presented as mean ± standard error of the mean. One-way analysis of variance (ANOVA) followed by Tukey posttest or 2-way ANOVA followed by Bonferroni posttest was used for statistical analysis for multiple groups. Nonnormally distributed data were transformed by Y=sqrt(Y+0.5) and ANOVA. *P* values < .05 were considered statistically significant.

## RESULTS

### *CLEC6A* (Dectin-2) Mutation

**Figure 1.**
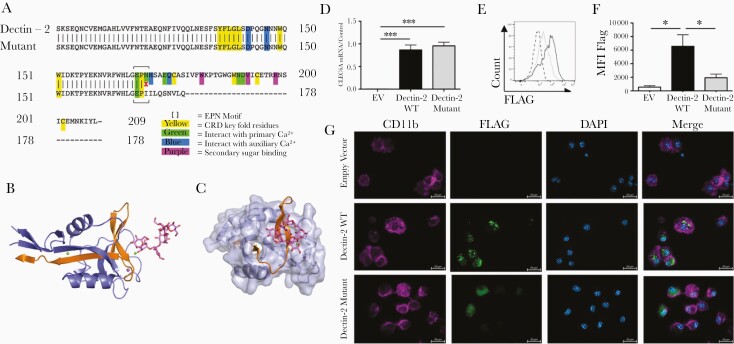
Dectin-2 mutation results in minimal protein expression. *A*, Partial amino acid sequence of wild-type (WT) and mutant (507delC) Dectin-2 with key residues and EPN mannan-binding motif highlighted. *B*, The fold of WT Dectin-2 5VYB (cartoon) in complex with mannan (stick model). Blue = present in the mutant protein, orange = absent in the mutant protein. Two Ca^2+^ atoms (green spheres) and an Na^+^ atom (purple) are also displayed. *C*, Predicted surface model of Dectin-2 covering the mutant structural elements only. The final β-strand at the core of the structure is missing in the mutant, resulting in a collapse of the motif, corruption of ligand interface, and inability to bind mannan. *D–G*, Bone marrow–derived macrophages (BMDMs) from Dectin-1–Dectin-2 knockout (KO) mice were infected with constructs expressing FLAG-tagged Dectin-2 WT, mutant, or empty vector (EV) and harvested 72 hours later. *D*, RNA was isolated, complementary DNA was prepared, and *CLEC6A* messenger RNA (mRNA) transcript was detected by reverse-transcription quantitative polymerase chain reaction. mRNA levels were normalized to *HPRT1*. Graph displays mean ± standard error of the mean (SEM) from 3 independent experiments. One-way analysis of variance (ANOVA) with Tukey posttest on transformed data. *E* and *F*, Cells were permeabilized, stained with anti-FLAG, and analyzed by flow cytometry. *E*, Dashed black line = empty vector; solid black line = Dectin-2; solid gray line = Dectin-2 mutant. Histogram representative of 3 independent experiments. *F*, Graph displays mean ± SEM of mean fluorescence intensity from 3 independent experiments. One-way ANOVA with Tukey posttest. *G*, BMDMs were stained with anti-CD11b (magenta) and anti-FLAG (green); nuclei were stained with 4’,6-diamidino-2-phenylindole (blue). Images are representative of 2 independent experiments. *P < .05; ***P < .001. Abbreviations: DAPI, 4’,6-diamidino-2-phenylindole; EV, empty vector; MFI, mean fluorescence intensity; mRNA, messenger RNA; WT, wild-type.

The patient possessed a homozygous base pair deletion (507delC) in exon 6 of Dectin-2 (*CLEC6A*) (Supplementary Figure 1*A*), which causes a frame shift (N170I) and premature termination of Dectin-2 (Figure 1A). Loss of Cys176 removes a disulphide bridge, while loss of Asp191 removes a Ca^2+^ and Na^+^ stabilizing bridge (Figure 1B) and loss of the final β-strand would leave a large hole at the core of the protein (Figure 1C), resulting in failure of the protein to fold. Furthermore, mutant Dectin-2 could not bind its ligand (Supplementary Figure 1*B* and 1*C*). Therefore, this Dectin-2 mutation likely has serious functional and clinical consequences.

Based on computational modeling, we hypothesized that mutant Dectin-2 would not produce a stable protein product. HEK293T cells expressing mutant Dectin-2 displayed increased RNA levels but minimal protein levels compared to WT Dectin-2 (Supplementary Figure 1*D*, 1*E* and 1*F*). Similarly, Dectin-1–Dectin-2 DKO BMDMs expressing mutant Dectin-2 displayed normal RNA levels (Figure 1D) but minimal protein levels compared to BMDMs expressing WT Dectin-2 (Figure 1E and 1F). Furthermore, while WT Dectin-2 clustered at/near the cell surface, mutant Dectin-2 was expressed at low levels throughout the cytosol and did not form clusters (Figure 1G and Supplementary Figure 1*G*). Together, these data indicate that Dectin-2 N170I does not form a stable protein product, is minimally expressed, and is therefore functionally defective.

### Functional Consequences of *CLEC6A* Mutation

**Figure 2.**
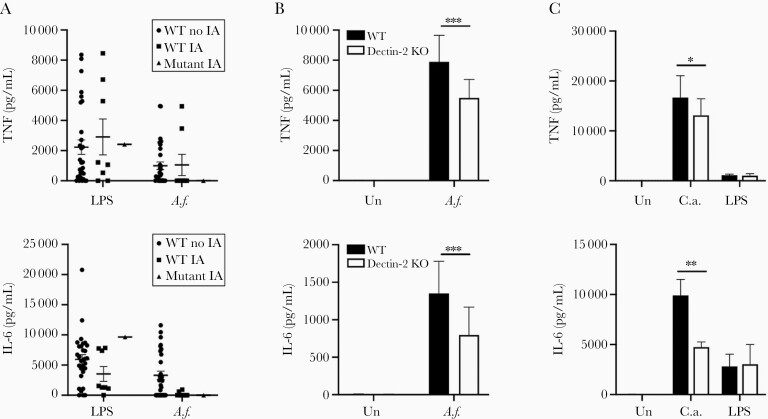
Dectin-2 mediates cytokine response to *Aspergillus fumigatus* and *Candida albicans*. *A*, Patient peripheral blood mononuclear cells were stimulated with *A. fumigatus* swollen conidia at a ratio of 1:1 or with lipopolysaccharide (LPS) for 24 hours. Cytokine levels in supernatants were measured by enzyme-linked immunosorbent assay (ELISA). Graphs show mean ± standard error of the mean (SEM) from 41 patients with wild-type (WT) Dectin-2 and 1 patient homozygous for mutant Dectin-2. Patient results are additionally stratified by their invasive aspergillosis status. *B*, Bone marrow–derived dendritic cells (BMDCs) from WT and Dectin-2 knockout (KO) mice were stimulated with *A. fumigatus* conidia at a ratio of 1:1 for 24 hours. Cytokine levels in supernatants were measured by ELISA. Graphs show mean ± SEM from 4 independent experiments, 2-way analysis of variance (ANOVA) on transformed data with Bonferroni posttest. *C*, BMDCs from WT and Dectin-2 KO mice were stimulated with *C. albicans* at a ratio of 1:1 or LPS for 24 hours. Amphotericin B was added after 2 hours and supernatants were harvested after 24 hours. Graphs show mean ± SEM from 3 independent experiments, 2-way ANOVA on transformed data with Bonferroni posttest. *P < .05; **P < .005; ***P < .001. Abbreviations: *A.f.*, *Aspergillus fumigatus*; *C.a.*, *Candida albicans*; IA, invasive aspergillosis; IL-6, interleukin 6; KO, knockout; LPS, lipopolysaccharide; TNF, tumor necrosis factor; Un, unstimulated; WT, wild-type.

To determine the functional consequences of Dectin-2 N170I, we tested whether the patients in our study were able to mount an effective immune response against *A. fumigatus*. Most WT PBMCs produced LPS- and *A. fumigatus*–induced cytokines. However, mutant Dectin-2 PBMCs only produced LPS-induced cytokines and not *A. fumigatus*–induced cytokines (Figure 2A). To confirm a role for Dectin-2 in *A. fumigatus*–induced cytokine production, we utilized Dectin-2 KO cells. Dectin-2 KO BMDCs displayed reduced *A. fumigatus*–induced (Figure 2B) and *C. albicans*–induced cytokine production compared to WT controls, whereas they displayed normal LPS-induced cytokine production (Figure 2C). However, Dectin-2 did not contribute to fungal killing (Supplementary Figure 2*A*).

We next investigated whether Dectin-2 mediated binding to *A. fumigatus*. Dectin-1–Dectin-2 DKO BMDMs expressing mutant Dectin-2 displayed a modest reduction in binding *A. fumigatus* compared to DKO BMDMs expressing WT Dectin-2 (Supplementary Figure 2*B* and 2*C*). *Aspergillus fumigatus*–induced trained immunity could be significantly reduced by blocking Dectin-2 in human monocytes (Supplementary Figure 2*D*). Together, these data indicate that Dectin-2 N170I has detrimental consequences for antifungal immunity. In agreement with this, the patient was diagnosed with probable IA and lung abnormalities consistent with fungal infection, which progressively worsened until death (Supplementary Table 2).

## Discussion

Herein, we characterized a novel Dectin-2 N170I mutation identified in an HSCT recipient who died of complications of IA. This mutation results in truncation of Dectin-2 and radically alters the receptor’s tertiary structure, leading to significantly reduced expression. Dectin-2 is important for fungal binding, trained immunity, and fungal-induced cytokine production.

Multiple polymorphisms in CLRs and their signaling component CARD9 increase susceptibility to fungal infection [9, 11], some even without immunosuppression [8]. Two patients with reduced CARD9 protein expression developed IA [12], and HSCT recipients with the Dectin-1 Y238X SNP, which results in a truncated CLR, displayed increased susceptibility to IA [9]. Here, we demonstrate that the Dectin-2 N170I mutation also results in a truncated CLR. The Dectin-2 mutation was identified in the recipient prior to SCT; however, up to 70% of tissue resident cells remain from host origin and may persist for up to 1 year [13–15]. The patient displayed signs of *A. fumigatus* infection <1 year post-HSCT, when host cells expressing mutant Dectin-2 were likely present in the lung. While the patient died of complications of IA, additional patients would be required to confirm a direct link between Dectin-2 N170I and IA.

Structural modeling of Dectin-2 N170I predicted incorrect folding of the protein. Consistent with this, we showed reduced expression of Dectin-2 N170I at the cell surface despite the presence of RNA and low level of dispersed intracellular protein. These results suggest that Dectin-2 N170I forms an unstable structure, is poorly transported to the cell membrane, and is minimally expressed, similar to Dectin-1 Y238X [8, 9]. Furthermore, Dectin-2 predominantly recognizes mannose, and hence *Aspergillus*, through its EPN motif, a structure lost in Dectin-2 N170I [4].

Dectin-2 generates robust cytokine and chemokine responses against *A. fumigatus* [4], and mice deficient in Dectin-2 are susceptible to *C. albicans* infection [5]. Here, we found a significant role for Dectin-2 mediating *A. fumigatus*– and *C. albicans*–induced tumor necrosis factor (TNF) and interleukin 6 (IL-6) secretion. Similarly, PBMCs from CARD9-deficient patients display impaired fungal-induced cytokine production [16], and mice with TNF blockade or IL-6 deficiency are highly susceptible to IA [17, 18]. Dectin-2 has previously been shown to bind to *A. fumigatus* hyphae [4], and we observed a modest reduction in binding of mutant Dectin-2 to *A. fumigatus* compared to WT Dectin-2. Importantly, we observed that Dectin-2 mediated *A. fumigatus*–induced trained immunity, further supporting the importance of Dectin-2 during IA.

Our research is the first to functionally characterize a Dectin-2 mutation associated with decreased antifungal responses. Furthermore, the Dectin-2 mutant patient developed and died of complications of IA. The Dectin-2 N170I mutation renders the CLR functionally null, and loss of Dectin-2 results in defective antifungal responses. Identifying mutations that increase a patient’s fungal susceptibility may permit a personalized approach and enable targeted prophylaxis of patients at high risk of fungal disease [11].

## Supplementary Data

Supplementary materials are available at *The Journal of Infectious Diseases* online. Consisting of data provided by the authors to benefit the reader, the posted materials are not copyedited and are the sole responsibility of the authors, so questions or comments should be addressed to the corresponding author.

jiab145_suppl_Supplementary_Figure_1

jiab145_suppl_Supplementary_Figure_2

jiab145_suppl_Supplementary_Material

jiab145_suppl_Supplementary_Table_1

jiab145_suppl_Supplementary_Table_2

## Notes

***Acknowledgments.*** S. J. O. was funded by a Sir Henry Dale Fellowship jointly funded by the Wellcome Trust and the Royal Society (grant number 099953/Z/12/Z) and by the T. Maelgwyn Davies Bequest fund. P. R. T. is supported by a Wellcome Trust Investigator Award (107964/Z/15/Z) and the United Kingdom Dementia Research Institute. E. S. was supported by a Medical Research Council GW4 BioMed DTP PhD studentship. M. A. C. is supported by a Biotechnology and Biological Sciences Research Council Discovery Fellowship (BB/T009543/1).

***Potential conflicts of interest.*** All authors: No reported conflicts of interest.
