# Supplementary figures and images for: A Human Dectin-2 Deficiency Associated With Invasive Aspergillosis
Source: J Infect Dis. 2021 Mar 18;224(7):1219–24. doi: 10.1093/infdis/jiab145 (PMC8514184; doi:10.1093/infdis/jiab145)

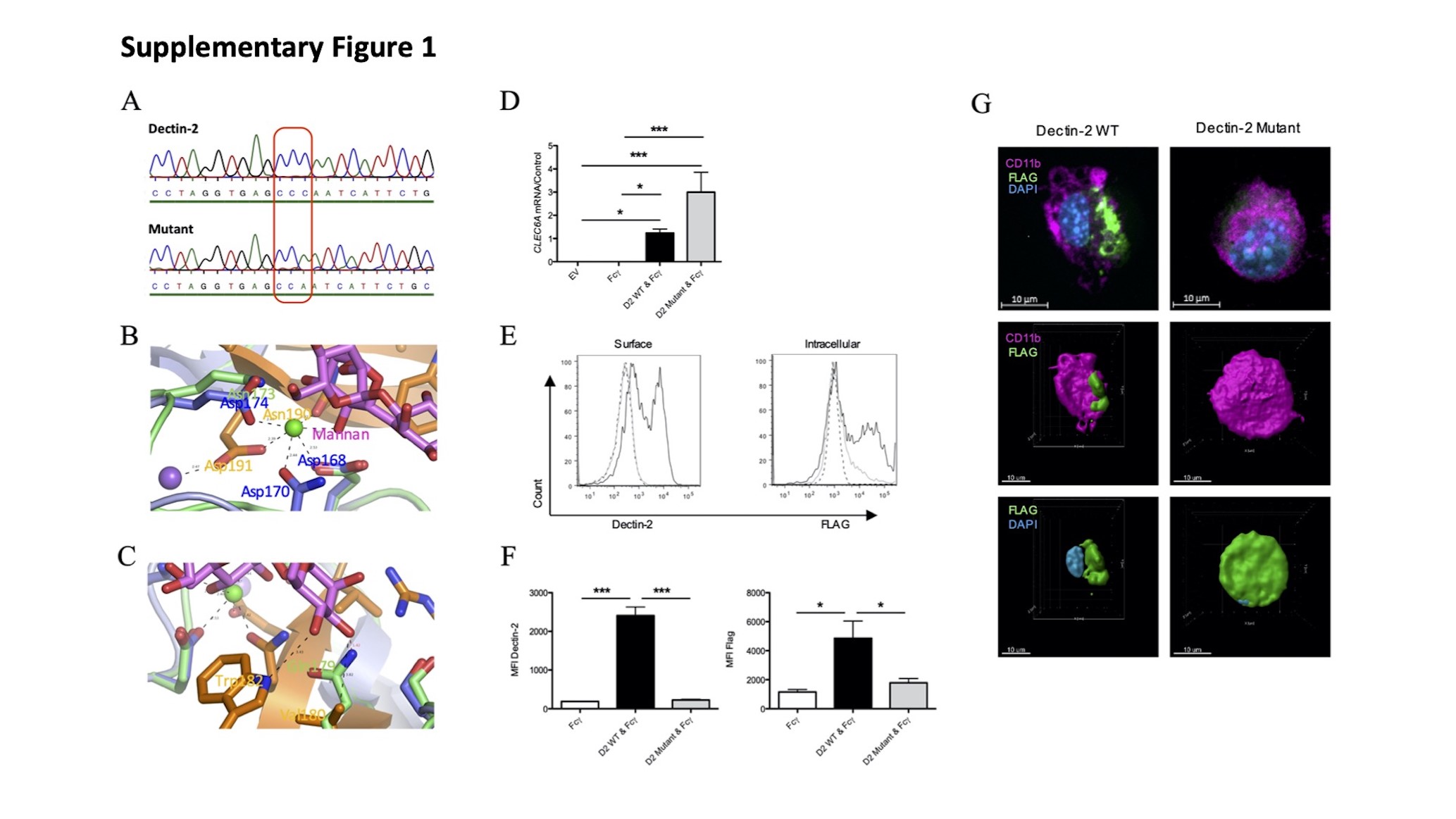

Supplement: jiab145_suppl_Supplementary_Figure_1 [file jiab145_suppl_supplementary_figure_1.jpeg]

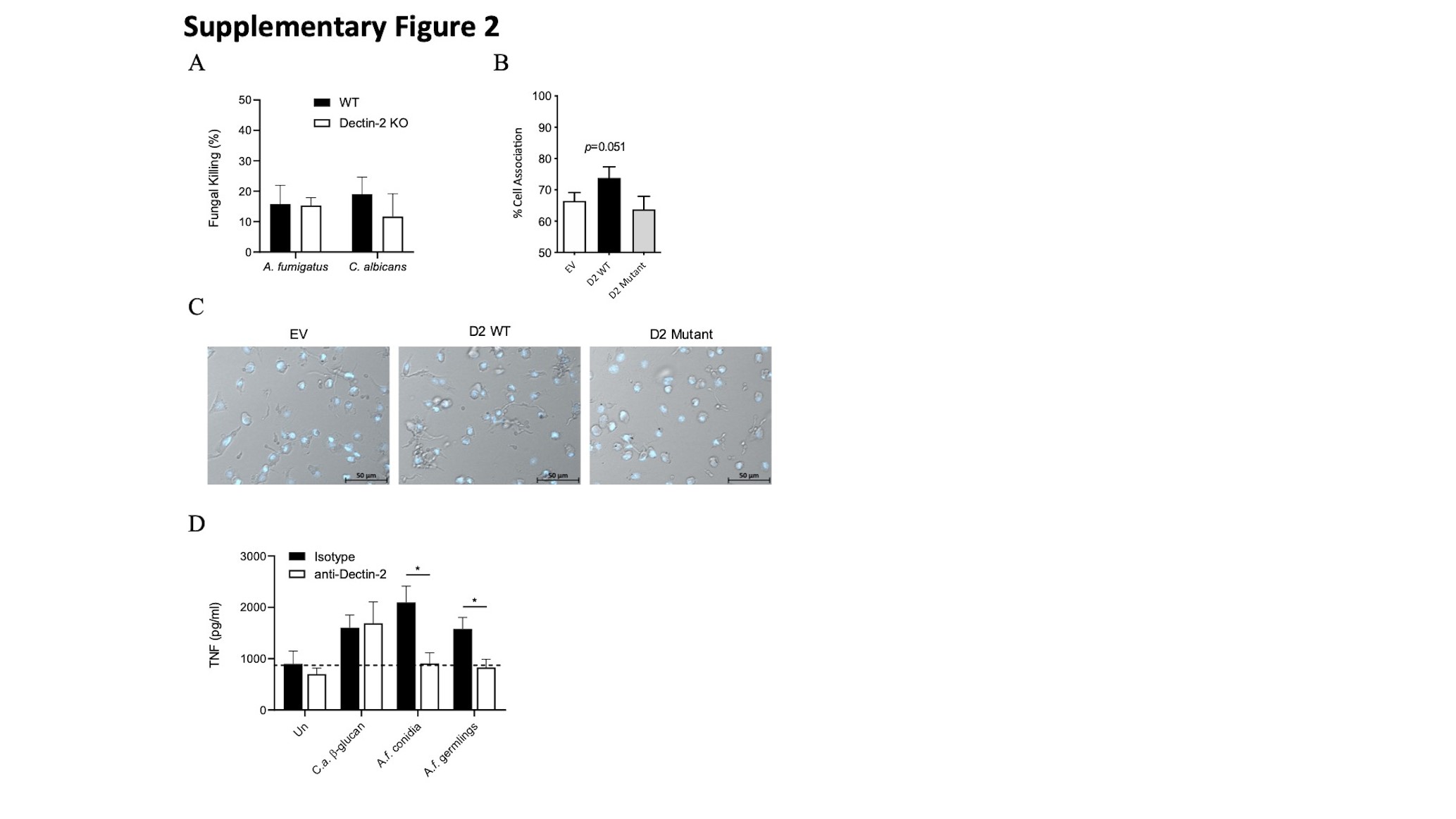

Supplement: jiab145_suppl_Supplementary_Figure_2 [file jiab145_suppl_supplementary_figure_2.jpeg]
