# Supplementary material for: A Human Dectin-2 Deficiency Associated With Invasive Aspergillosis
Source: J Infect Dis. 2021 Mar 18;224(7):1219–24. doi: 10.1093/infdis/jiab145 (PMC8514184; doi:10.1093/infdis/jiab145)
Supplement: jiab145_suppl_Supplementary_Material [file jiab145_suppl_supplementary_material.docx]

**SUPPLEMENTARY APPENDIX**

**A Human Dectin-2 Deficiency Associated With Invasive Aspergillosis**

James S. Griffiths^1,2^, P. Lewis White^3^, Magdalena A. Czubala^1^, Elena Simonazzi^1,4^, Mariolina Bruno^5^, Aiysha Thompson^1,4^, Pierre J. Rizkallah^1^, Mark Gurney^1^, Diogo M. da Fonseca^1^, Julian R. Naglik^2^, Wendy Ingram^6^, Keith Wilson^6^, Frank L. van de Veerdonk^5^, Rosemary Barnes^6^, Philip R. Taylor^1,4^, Selinda J. Orr^1,7^.

**SUPPLEMENTARY METHODS**

**Patient Samples**

Written informed consent was obtained from all patients in the study. A prospective cohort of 42 patients with hematological malignancies admitted for stem cell transplantation (SCT) (23 patients) or remission-induction therapy for acute myeloid leukemia (AML) (19 patients) were recruited. Of the 23 SCT patients included in this study, 5 developed proven IA and 1 developed probable IA. Of the 19 AML patients included in this study, 4 developed proven IA. Clinical information for the patient with the 507DelC Dectin-2 mutation can be found in Supplementary Table 1. To identify the ability of high-risk patients to mount an effective immune response to *A. fumigatus*, blood samples were collected on admission to hospital from patients. Blood samples were collected in PAXgene blood RNA tubes (Qiagen) and in EDTA blood collection tubes (Becton Dickinson).

**Genetic Analysis**

Dectin-2 DNA was amplified from patient cDNA by PCR using the primers specified in Supplementary Table 1. Four Dectin-2 primers were used in sequencing reactions to provide appropriate Dectin-2 coverage. These primers are described in Supplementary Table 1. Dectin-2 sequencing was completed at GATC Biotech, Germany. Following identification of the 507delC/N170I mutation, the above process was repeated from this patient’s RNA sample to confirm. The patient’s second round of sequencing was completed at Eurofins Genomics, Luxembourg.

**Modeling of Dectin-2 Structure**

The structure of wild type Dectin-2 [1], PDB accession code 5VYB, was used as the starting model. COOT was used to implement the mutations and readjustment of the model. REFMAC5 from the CCP4 package was used to regularize the geometry of the mutant model. No energy minimization was conducted, because the large missing stretch of the protein chain after the mutation, would have caused a collapse in the fold of the protein structure, which is beyond the scope of the present exercise. Graphics illustrations were prepared with PYMOL.

**Lentivirus titre**

Where Lentivirus was used, Lentivirus titre was verified using Jurkat cells. 2x10^5 Jurkat were seeded in 24 well plate in RPMI1640 (supplemented with 10% FBS, 100 U/ml penicillin and 10µg/ml streptomycin) and infected with multiple doses of lentivirus ranging from 0.25 to 10µl. After 3 days, FLAG-tag expression was measured using flow cytometry in Jurkat cells fixed with 2% PFA.

**Fungal Killing Assay**

WT and Dectin-2 KO BMDCs were resuspended in RPMI containing 10% FBS and 100U/ml penicillin/streptomycin. 100μl of 1x10^6^ BMDCs/ml were challenged with 100μl of 1x10^6^/ml *A. fumigatus* conidia or 100μl of 1x10^6^/ml *C. albicans*, centrifuged at 350g for 5 minutes and incubated at 37^o^C. After 3 h well contents were collected and lysed with 1% (v/v) Triton X-100 in PBS, lysed contents were spotted onto PDA plates and CFU determined after 24 h.

**Trained Immunity Assay**

Buffy coats from healthy donors were obtained after they provided written informed consent (Sanquin Blood Bank, Nijmegen, The Netherlands) and PBMCs were isolated. Briefly, patient blood was diluted in PBS (1:1) and fractions were separated by Ficoll (GE Healthcare). PBMCs were washed three times with PBS before Percoll isolation of monocytes was performed. Briefly, 150-200x10^6^ PBMCs were layered on top of a hyper-osmotic Percoll solution (48,5% Percoll, 41,5% sterile H_2_O, 0.16M filter sterilized NaCl) and centrifuged for 15 minutes at 580 g. The interphase layer was isolated, and cells were washed with cold PBS. Cells were resuspended in RPMI 1640 Dutch modification culture medium supplemented with 10 μg/ml gentamicin (Centraform), 2 mM glutamax (Invitrogen), and 1 mM pyruvate (Invitrogen) and counted, adjusting the concentration to 1 × 10^6^/mL.

Monocytes were trained as previously described [2]. Briefly, 100,000 cells were added to flat-bottom 96-well plates (Corning), adhered for 1 h at 37°C and gently washed with warm PBS to yield maximal purity. Monocytes were pre-incubated for 1 h with either 10μg/mL anti-Dectin-2 antibody (MAB3114 Bio-Techne/R&D) or 10 μg/mL of its control IgG1 isotype (MAB002 Bio-Techne/R&D). After 1 h, cells were incubated either with culture medium only, or with 1μg/ml of *C. albicans* β-1,3-(D)-glucan (kindly provided by Professor David Williams, College of Medicine, Johnson City, USA), 1x10^6^/mL heat-inactivated *A. fumigatus* conidia and swollen conidia (V-05, clinical isolate) or 10μg/ml of *A. fumigatus* *α*-(1→3) glucan (kindly provided by Vishukumar Aimanianda, Institute Pasteur, Paris) for 24h in 10% pooled human serum. After 24h, the plates were washed twice with 200 μL of warm PBS, and medium with 10% pooled human serum was added for 5 days and refreshed after two to three days. In these five days, monocytes differentiated towards macrophages, and at day six cells were re-stimulated with or without 10ng/ml LPS (Sigma). After 24h supernatants were collected, and cytokine concentrations measured by ELISA (R&D Systems).

**SUPPLEMENTARY RESULTS**

**Supplementary Figure 1: Dectin-2 mutation results in abnormal protein localisation and reduced expression.** (A) The exonic regions of Dectin-2 (*CLEC6A*) were sequenced. In one patient a homozygous nucleotide deletion (507delC) in exon 6 of Dectin-2 was observed. The sequencing chromatographs covering this region for a patient with the functional wild type Dectin-2 receptor is compared to the patient with the homozygous deletion mutation. The Dectin-2 mutant patient had no other sequencing or expression abnormalities in Dectin-1 *(CLEC7A)*, Mincle *(CLEC4E)* and Mcl *(CLEC4D)*. Additionally, the presence of a robust LPS response suggests a functioning TLR4 pathway [5]. (B-C) The contact point between Dectin-2 and mannan. The structure in blue is present in the mutant protein, the structure in orange is absent in the mutant protein. (B) After the loss of one codon, Asp174 becomes Asn173 and rotates away from the position of the Ca^2+^. Two other Ca^2+^ ligands, Asn190 and Asp191, are lost. Therefore, the Ca^2+^ ion cannot be bound and the anchor for the mannan is lost. (C) At the minor interface between WT Dectin-2 and mannan, Trp182 provides a weak, stabilising contact to the mannan and Val180 offers a vdW cushion completing the protective environment around mannan. Both these residues are absent in mutant Dectin-2. (D-F) HEK293T cells were transfected with constructs expressing FLAG-tagged Dectin-2 WT or mutant alongside the Fc$\gamma$R signalling chain or empty vector. Cells were harvested 48 h later. (D) RNA was isolated, cDNA was prepared and *CLEC6A* (Dectin-2) mRNA transcript was detected by RTqPCR. mRNA levels were normalized to *HPRT1*. Graph displays mean +/- SEM from 3 independent experiments. One-way ANOVA with Tukey’s post-test on transformed data. (E-F) Cells were surface stained with anti-Dectin-2 or intracellularly stained with anti-FLAG and analysed by flow cytometry. (E) Dashed black line = empty vector, solid black line = Dectin-2, solid grey line = Dectin-2 mutant. Histograms are representative of 3 independent experiments. (F) Graphs display mean +/- SEM mean fluorescent intensity (MFI) from 3 independent experiments. One-way ANOVA with Tukey’s post-test. (G) BMDMs from Dectin-1-Dectin-2 DKO mice were infected with constructs expressing FLAG-tagged Dectin-2 WT or mutant and harvested 72 h later. Cells were stained with anti-CD11b (magenta) and anti-FLAG (green) and nuclei were stained with DAPI (blue). Z-stacks of the entire cellular volume were obtained with a confocal microscope and used to generate 3D images with IMARIS software. Images are representative of 2 independent experiments.

**Supplementary Figure 2: Dectin-2 mutation modestly reduces *A. fumigatus* binding and significantly reduces *A. fumigatus*-specific trained immunity.** (A) BMDCs from WT or Dectin-2 KO mice were stimulated with A *fumigatus* or C. *albicans* for 3 h before BMDCs were lysed and lysates spotted onto PDA plates. Fungal killing was determined against a control sample containing only fungi. Graph displays mean +/- s.e.m. from 5 independent experiments, two-way repeated measures ANOVA with Sidak’s post-test. (B-C) BMDMs from Dectin-1-Dectin-2 DKO mice were infected with constructs expressing FLAG-tagged Dectin-2 WT or mutant or empty vector. 72 h later cells were incubated with *A. fumigatus* early germlings for 2 h. Cell nuclei were stained with DAPI (blue) and cells were imaged with a Zeiss Cell Observer spinning disk confocal microscope. (B) Cell association was quantified by counting the total number of BMDMs in a field and then counting the number of BMDMs associated with (touching) *A. fumigatus*. The percentage cell association was calculated by dividing the number of BMDMs associated with *A. fumigatus* by the total number of cells in a field. 10 different fields were counted for each experiment. Graph displays mean +/- s.e.m. from 4 independent experiments, one-way ANOVA. (C) Images are representative of 4 independent experiments. (D) Human primary monocytes were either unstimulated or stimulated with *C. albicans* β-glucan (as Dectin-2 does not bind β-glucan this acted as a control), *A. fumigatus* conidia or *A. fumigatus* germlings in the presence of Dectin-2 neutralising antibody or its isotype for 24 h. After 24 h, all antibody and stimulants were removed. At day 6, cells were re-stimulated with LPS for 24 h before cytokine levels in supernatant were measured by ELISA. Graph displays mean +/- s.e.m. from 2 independent experiments each comprising of 3 different donor samples, Wilcoxon test on matched pairs. A Wilcoxon test on matched pairs was used to compare +/- treatment in Supplementary fig 2D

**Supplementary Table 1: Plasmids, Constructs and Primers used in this research.**

**Supplementary Table 2: Clinical details for patient with Dectin-2 507delC mutation.**

**SUPPLEMENTARY REFERENCES**

1. Feinberg, H., et al., *Mechanism of pathogen recognition by human dectin-2*. J Biol Chem, 2017. **292**(32): p. 13402-13414.

2. Bekkering, S., et al., *In Vitro Experimental Model of Trained Innate Immunity in Human Primary Monocytes*. Clin Vaccine Immunol, 2016. **23**(12): p. 926-933.

3. Demaison, C., et al., *High-level transduction and gene expression in hematopoietic repopulating cells using a human immunodeficiency virus type 1-based lentiviral vector containing an internal spleen focus forming virus promoter*. Hum Gene Ther, 2002. **13**(7): p. 803-13.

4. Kitamura, T., *New experimental approaches in retrovirus-mediated expression screening*. Int J Hematol, 1998. **67**(4): p. 351-9.

5. Chow, J.C., et al., *Toll-like receptor-4 mediates lipopolysaccharide-induced signal transduction*. J Biol Chem, 1999. **274**(16): p. 10689-92.
