# Supplementary material for: A Human Dectin-2 Deficiency Associated With Invasive Aspergillosis
Source: J Infect Dis. 2021 Mar 18;224(7):1219–24. doi: 10.1093/infdis/jiab145 (PMC8514184; doi:10.1093/infdis/jiab145)
Supplement: jiab145_suppl_Supplementary_Table_1 [file jiab145_suppl_supplementary_table_1.docx]

**Supplementary Table 1: Plasmids, Constructs and Primers used in this research.**

| **Plasmids and constructs used in this study** | **Reference** | |
| --- | --- | --- |
| pHR’SIN-cPPT-SXW | **[3]** | |
| FcγR pMXs-IP | **[4]** | |
| **Amplifying and sequencing Dectin-2 in Patient Samples** | **Primer** | **Use** |
| ATGATGCAAGAGCAGCAA | Forward Primer 1 | Amplifying and sequencing  Dectin-2 |
| TCATAGGTAAATCTTATTCATCTCACAT | Reverse Primer 1 |  |
| CCCAGCTTCTTGGAAGTCA | Forward Primer 2 | Sequencing Dectin-2 |
| ACTCATTCAGCTGCTGGAC | Reverse Primer 2 |  |
| **Cloning Dectin-2 pFB-NEO constructs** | **Primer** | **Use** |
| ATGGACTACAAAGACGATGACGACAAGATGCAAGAGCAGCAACC | N-T WT & Mut STEP 1 Forward | Add KOZAK and N-terminal FLAG tag to Dectin-2 and sequencing Dectin-2 |
| TCATAGGTAAATCTTATTCATCTCACAT | N-T WT & Mut STEP 1 Reverse |  |
| CGACGAATTCGGATCCCACCATGGACTACAAAGAC | N-T WT & Mut STEP 2 Forward | Inserting N-terminal FLAG tag Dectin-2 into pFB-NEO at BamH1 site using Infusion and sequencing Dectin-2 |
| GGCCGCTCGAGGATCTCATAGGTAAATCTTATTCATCTCA | N-T WT & Mut STEP 2 Reverse |  |
| **Cloning Dectin-2 pSXW constructs** | **Primer** | **Use** |
| GGATCCCGGGCTCGAGCCACCATGGACTACAAAGACGATGAC | Dec2 Infusion-F | Inserting N-terminal FLAG tag Dectin-2 into pSXW at Xho1 site using Infusion |
| TACCAGGCCTCTCGAGTCATAGGTAAATCTTATTCATCTCA | Dec2 SXW Infusion-R |  |
| AGGCCAAGAACAGATGGTCC | Seq-Forward | Sequencing Dectin-2 |
| GAAGTTCATCTGCACCACCG | Seq-Reverse 1 |  |
| CACTGTGTTTGCTGACGCAACC | Seq-Reverse 2 |  |
