# Supplementary material for: A Human Dectin-2 Deficiency Associated With Invasive Aspergillosis
Source: J Infect Dis. 2021 Mar 18;224(7):1219–24. doi: 10.1093/infdis/jiab145 (PMC8514184; doi:10.1093/infdis/jiab145)
Supplement: jiab145_suppl_Supplementary_Table_2 [file jiab145_suppl_supplementary_table_2.docx]

**Supplementary Table 2: Clinical details for patient with Dectin-2 507delC mutation**

| **Characteristic** | **Day** | **Patient** |
| --- | --- | --- |
| **Dectin-2 nucleotide genotype** |  | I/I |
| **Age** |  | 41 |
| **Primary Disease** |  | Acute Lymphoblastic Leukemia |
| **Treatment** |  | Allogeneic SCT with fluconazole post-transplant (no mold prophylaxis as local policy to screen and treat pre-emptively) |
| **Infections** |  | CMV reactivation, invasive aspergillosis and parainfluenza |
| **Timeline of infections** | -419  -413  -313  -176  -78  -71  -71  -64  -29  -15  -14  0 | Sample collection (Dectin-2 mutation detected in this sample)  Allogeneic SCT that engrafted 10 days later, no further neutropenia, no GVHD  Chronic upper respiratory tract infection (first suggestion of *A. fumigatus* infection) and hearing loss  *A. fumigatus* PCR positive and galactomannan (GM) ELISA positive (first biomarkers for *A. fumigatus*)  Parainfluenza type 4 positive by PCR  Chest CT showed bilateral nodularity/ground glass opacification of lungs suggestive of fungal infection (given voriconazole anti-fungal treatment from this point on)  Fever/cough detected  *A. fumigatus* cultured from sputum, but *A. fumigatus* negative by PCR and GM ELISA (biomarkers negative)  Repeat chest CT showed progression of radiological appearances consistent with fungal infection (diagnosed as probable IA at this point)  Parainfluenza type 4 negative by PCR  Repeat chest CT showed further progression of radiological appearances consistent with fungal infection  Time of death, invasive aspergillosis associated cause of death |
